# Aberrant emotion networks in early major depressive disorder patients: an eigenvector centrality mapping study

**DOI:** 10.1038/tp.2016.81

**Published:** 2016-05-24

**Authors:** Z Song, M Zhang, P Huang

**Affiliations:** 1 Department of Neurology, Binjiang Hospital, 2nd Affiliated Hospital, Zhejiang University School of Medicine, Hangzhou, China; 2 Department of Neurology, 2nd Affiliated Hospital, Zhejiang University School of Medicine, Hangzhou, China; 3 Department of Radiology, 2nd Affiliated Hospital, Zhejiang University School of Medicine, Hangzhou, China

## Abstract

Major depressive disorder (MDD) is a serious mental disorder that negatively affects the quality of life of many individuals, and is a heavy economic burden to society. In recent years it was thought that depression is a ‘disconnection syndrome'. Disorganized brain activity and un-modulated emotion responses were considered the key neuropathologies underlying depression. In the present study, we investigated the alteration of whole brain network connectivity in 28 first-episode, drug-naive patients, using resting-state functional magnetic resonance imaging and a new analytical method called voxel-based eigenvector centrality mapping. We found that compared with normal controls, MDD patients had lower functional connectivity in the bilateral middle frontal gyrus, insula, hippocampus, amygdala and cerebellum, and higher functional connectivity in the medial prefrontal cortex. The functional connectivity strength at the right hippocampus (*r*=−0.413, *P*=0.032) and the right insula (*r*=−0.372, *P*=0.041) negatively correlated with the severity of the disease. We further examined coordination among these regions, and found that frontal–subcortical connection was reduced and insula–mPFC connection was increased. These results are consistent with previous hypotheses on the neural mechanism of MDD, and provide further evidence that emotion networks are already interrupted in early stages of depression.

## Introduction

Major depressive disorder (MDD) is a serious psychiatric mood disorder, which typically presents as persistent dysthymia, anhedonia and occasional suicidal ideation and behaviors.^1^ The lifetime prevalence of MDD ranges from 1.5^2^ to 16.2%^3^ across various studies. MDD exerts a heavy socioeconomic burden because of increased disability and suicide rates.^4^ The neuropathology underlying MDD is still unclear. Therefore, even at present, the diagnosis and treatment of depression can be challenging.

In recent years, it has been suggested that depression is not localized to a single brain area and that a network perspective is necessary to explain its complex etiology. Based on the fact that widespread brain areas were found abnormal in MDD patients,^5, 6^ constituting an ‘emotion network', more and more researchers have begun to believe that depression is a ‘disconnection syndrome'.^7, 8^ Alteration in important nodes of the emotion network may induce aberrant function of the whole network, causing depression. Specifically, it has been suggested that frontal–subcortical neural circuits play an important role in the pathogenesis of MDD.^9^ Interrupted coordination within and among large-scale functional networks were also implicated.^10, 11^ Both gray matter and white matter deficits in these populations might be the reason for disrupted network functions.^12, 13, 14^ Indeed, a network perspective supports the high heterogeneity of depression, and explains how different treatment methods might take effect.^15^

Although these hypotheses have found support from neuroimaging-based network studies, inconsistency still exists, possibly due to the different methods used in these studies. In the past, seed-based functional connectivity had been extensively used to explore the coordination between a specific region of interest (ROI) and the whole brain, or among several pre-specified ROIs. While it is useful to test a particular hypothesis, the selection of ROIs is quite subjective. As different researchers use different ROIs, it is difficult to make direct comparisons across studies.^16, 17^ In addition, the selected ROIs may not be truly representative of the designated region because of segmentation problems^18^ or template bias^19^ or high heterogeneity within them. Different ROI selection methods may greatly influence the result of network connectivity analysis.^20^ This problem also exists in ROI-based whole brain graph theory analysis.^21^ Despite the fact that some data-driven methods were used, such as independent component analysis, they usually explain sub-network connectivity^10^ or interaction among large-scale functional networks.^22^ Therefore, a simple voxel-based, bias-free method is desirable for future studies and clinical applications assessing brain network changes in MDD patients.

In the present study, we aimed to assess the brain network alterations in MDD patients, using resting-state functional magnetic resonance imaging and a new graph-based method called eigenvector centrality mapping (ECM). This method can objectively detect all the brain areas serving as communication hubs, which have greater connectivity with other parts of the brain. It can be performed in a voxel-wise manner and does not require manual selection of the seed regions, therefore it is free of researcher selection bias. We hypothesize that emotion-related networks may have already been interrupted in early MDD patients, and these alterations can be detected using ECM.

## Materials and methods

### Subjects

We recruited 28 MDD patients in the present study. Unipolar depression was diagnosed according to DSM-IV criteria by an experienced psychiatrist. Scores of the Hamilton Rating Scale for Depression (HRSD), Beck Depression Index (BDI) and the Mini-Mental State Examination (MMSE) were obtained from all subjects. Twenty-seven normal controls (NC) without any history of neurologic or psychiatric disorders, or brain trauma at any time of their lives, were also enrolled. Table 1 shows detailed characteristics of the two groups. All patients were first-episode, drug-naive patients with short disease durations. All the subjects signed written informed consent before taking part in the study. This research was approved by the Medical Ethic Committee of the Second Affiliated Hospital, Zhejiang University School of Medicine.

### fMRI data acquisition

Scanning was carried out on a 3.0 T magnetic resonance scanner (Siemens 3.0T Trio Tim, Munich, Germany). Participants were fitted with soft earplugs, positioned comfortably in the coil, and instructed to relax and remain still. Head motion was minimized with foam pads. Participants were excluded if they had excessive head motion with >2 mm of translation or 2° of rotation, which was confirmed on a workstation immediately after the scanning. High-resolution three-dimensional T1-weighted images were acquired with a three-dimensional magnetization-prepared rapid acquisition gradient echo sequence with the following parameters: repetition time 8.5 ms, echo time 3.4 ms, flip angle 12°, 156 axial slices with 1 mm thickness, axial field of view 24 × 24 cm^2^ and matrix 256 × 256. Blood oxygen level-dependent images were acquired using an echo planar imaging sequence. The acquisition parameters were: time of repetition=2000 ms, time of echo=30 ms, flip angle=90°, 30 interleaved descending slices and voxel size=3.8 × 3.8 × 5.0 mm^3^. Several other sequences were also scanned and the total duration for each subject lasted about 40 min.

### Preprocessing

Preprocessing was performed using the Data Processing Assistant for Resting-State fMRI (http://www.restfmri.net) and the Resting-State fMRI Data Analysis Toolkit (Rest, V1.8, http://www.restfmri.net), based on statistical parametric mapping-8 (SPM8, www.fil.ion.ucl.ac.uk/spm/) and Matlab R2010 (www.mathworks.com). The first 10 images were excluded from the analysis. The remaining images were corrected for slice timing with the middle slice used as a reference, realigned to remove head motion, normalized into the standard space, and resampled to a 3 × 3 × 3 mm^3^ voxel size. The resulting images were then smoothed using a 4-mm Gaussian kernel before proceeding to the next step.

### Functional connectivity analysis

The human brain is organized as a complex network with small world properties.^23^ Therefore, graph-based analysis could provide valuable information for elucidating the brain's network structures. Eigenvector centrality is a particular type of graph-based method that identifies important nodes in the network. It does so by counting both the number and the quality of connections so that a node with few connections to some other high-ranking nodes may outrank one with a larger number of low-ranking connections.^24^ Google's ‘PageRank' algorithm^25^ is a variant of eigenvector centrality. Like its success in the web search engine, eigenvector centrality has also been proven to be valuable in analyzing human brain networks.^26, 27^

Here, the ECM of the pre-processed image data was performed using the fast ECM tool (https://code.google.com/p/bias/source/browse/matlab/fastECM), which yielded a voxel-wise measure of relevance to the functional brain network. Compared with the traditional ECM calculation method, the fast ECM tool is faster and computationally more efficient because it computes matrix-vector products without having to compute or store the connectivity matrix.^28^ Detailed analysis procedures can be seen in the study by Wink *et al.*^28^

By using ECM, we successfully identified several brain regions where there was a significant difference of nodal centrality. To further explore the relationship among these areas, and to test whether these findings were consistent with previous theories of the neural mechanism of depression, we calculated the functional connectivity among them. Blood oxygen level-dependent signals were extracted from all significant clusters, and Pearson correlation was calculated in a pair-wise manner. The correlation coefficients were transformed into *z* distribution using Fisher *r*-to-*z* transformation.

### Statistical analysis

Age was compared between the two groups using a two-sample *t*-test. A *X*^2^ test was performed to compare the sex differences. For image data, motion correction was assessed using frame wise displacement (FD), which measures the motion of each brain volume compared with the previous volume.^29^ Mean FD was compared between the two groups using an independent two-sample *t*-test. Eigenvector centrality differences between the two groups were compared using a two-sample *t*-test performed in SPM8, during which age and sex were included as covariates. The threshold for ECM analysis was set at *P*<0.001, voxel size >10, corrected using the false discovery rate method. Significant clusters were used to extract connectivity values from the two groups and to calculate correlation with scale scores. For connectivities among significant clusters, a two-sample *t*-test was used to examine the difference between the two groups. Multiple comparison correction was performed using the Bonferroni method.

## Results

**Figure 1:**
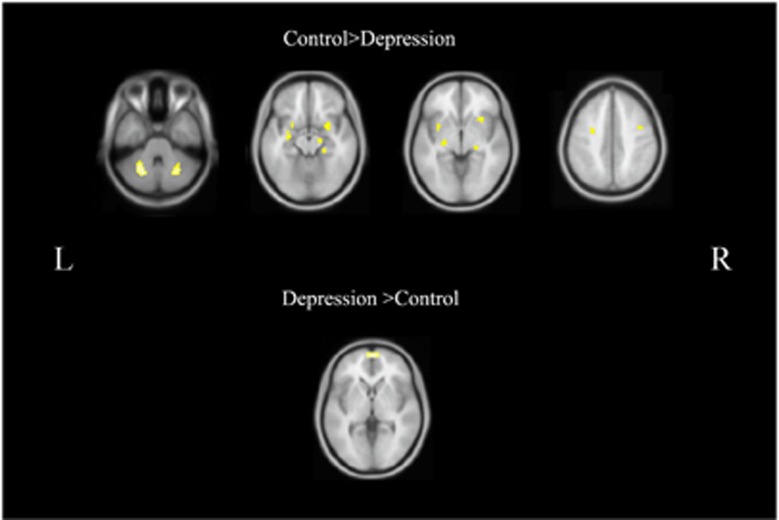
Functional connectivity strength differences between the MDD patients and normal controls. Warm colors indicate areas with higher functional connectivity in normal controls. Cold colors indicate higher functional connectivity in patients. MDD, major depressive disorder.

**Figure 2:**
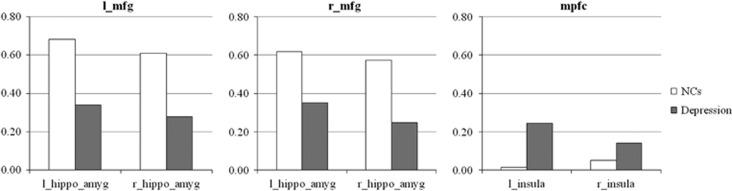
Frontal–subcortical and mPFC–insula functional connectivities of the two groups. Frontal–subcortical connections were significantly reduced in the patient group. The connection between left insula and mPFC significantly increased in the patients group. hippo_amyg, hippocampus and amygdala; l, left; mfg, left middle frontal gyrus; mPFC, medial prefrontal cortex; r, right.

Statistical analysis showed no significant differences in age and sex between the two groups. Analysis of mean FD demonstrated no significant differences between groups. ECM analysis showed that compared with NCs, the patients groups had lower functional connectivity in the bilateral middle frontal gyrus (MFG), insula, hippocampus, amygdala and cerebellum (Figure 1, Table 2). Meanwhile, the patients had increased functional connectivity in the mPFC. The effect size of this comparison was quite high, ranging from 1.33 to 1.69 for each significant cluster. Furthermore, functional connectivity strength at the right hippocampus (*r*=−0.413, *P*=0.032) and the right insula (*r*=−0.372, *P*=0.041) negatively correlated with the severity of the disease (HRSD). To test whether these alterations were in line with previous hypotheses about depression, we explored the frontal–subcortical connectivity and insula–mPFC connectivity. Six connections were calculated and compared between the two groups. As shown in Figure 2, we found that frontal–subcortical connections were significantly reduced in the patient group (Table 3). In contrast, the connection between left insula and mPFC was significantly increased in the patients group (*P*=0.003).

## Discussion

To the best of our knowledge, this is the first study using the voxel-based ECM method to assess the whole brain network connectivity of MDD patients. Compared with NCs, depressed patients showed decreased functional connectivity in the frontal lobes, amygdala, hippocampus and cerebellum, and increased functional connectivity in the mPFC. Correlation between functional connectivity strength and HRSD scores was found in the right hippocampus and right insula. Furthermore, we verified aberrant frontal–subcortical coordination and insula–default mode network (DMN) connection in the patient group, which is consistent with previous theories about the mechanism of depression. These results suggest that without choosing ROIs subjectively, we can still detect important emotion network changes in depressed patients using the ECM method, which may have great potential in future studies on network changes of MDD.

First, we found decreased functional connectivity in the bilateral MFG; part of the dorsolateral prefrontal cortex (DLPFC). The DLPFC is primarily responsible for cognitive and executive functions, though it also plays important roles in regulating emotions through reappraisal/suppression strategies.^30^ Abnormal DLPFC structures and functions have been consistently reported in early MDD patients^31, 32^ and those with familial risk for MDD.^33, 34^ It is believed that deficits in the DLPFC may confer vulnerability to depression.^35^ Here the decreased functional connectivity in the DLPFC implies that its interaction with other brain areas is weakened. The disruption of DLPFC connections has also been documented in several previous studies^36, 37^ using ROI-based functional connectivity method.

Second, we found decreased functional connectivity in the limbic regions, including amygdala and hippocampus. Limbic structures are very important for spontaneous response to external emotional information. However, the reappraisal and regulation of spontaneous activities in these areas rely on cognitive analysis in higher cortices. Depressed patients often have chaotic and disorganized limbic activities,^38, 39^ which may be why they more often show uncategorized, biased responses than normal subjects when facing emotional stimuli.^40^ Besides, there were negative correlations between the functional connectivity strength of the amygdala, hippocampus and HRSD scores, suggesting that the more disorganized they were, the greater the chance the disease would become worse.

Taken together, the decreased frontal–subcortical functional connectivity is consistent with the previous hypothesis about the frontal–subcortical uncoupling in depressed patients.^41, 42^ When the higher cognitive functions of the DLPFC were damaged, or frontal–subcortical connections were interrupted, activities in subcortical areas were no longer constrained, and became chaotic and blunted. This hypothesis is quite popular and has been tested in several studies using functional imaging methods to study the mechanism of MDD.^37, 43, 44^ A recent effective connectivity study showed that top–down regulation from the DLPFC to the amygdala was greatly impaired in depressed patients and bottom–up connections were increased.^37^ To explore whether the changes we found were in line with this hypothesis, we tested the connectivity among bilateral MFG and subcortical structures. The result showed significantly decreased connectivity between the frontal clusters and subcortical areas in depressed patients, confirming frontal–subcortical uncoupling.

Insula connectivity was found to be reduced. The insula also has a well-established role in processing affect and emotion, and has been frequently found to be abnormal in depressed patients.^45, 46^ Specifically, the anterior insula has rich connections with limbic and cortical regions. Together with the anterior cingulate gyrus, they form an important network called the salience network (SN). The SN is of specific importance for the processing of a stimulus salience^47^ and regulating balance between the DMN and executive control network,^48^ drawing much attention from researchers worldwide. As part of the SN, disturbance in the insula may greatly impair its ability in coordinating the patient's attention being directed either toward the external world or the internal perception of self-related processes, resulting in depressive symptoms. A previous study also found decreased anterior insula connectivity, which negatively correlated with disease severity.^49^

Interestingly, besides decreased functional connectivity, we also found increased functional connectivity in the medial prefrontal cortex of the depressed patients.^50^ The medial prefrontal cortex is a central hub of the DMN, which has an important role in introspection. The increased functional connectivity strength within the DMN has been consistently reported in studies on depression,^51, 52^ which could lead to the patient's clinical symptoms, such as rumination.^53^ Sheline *et al.*^54^ reported that depressed patients were characterized by the inability to reduce DMN activity when facing negative stimuli. Using independent component analysis, Zhu *et al.*^55^ also revealed increased functional connectivity in anterior medial regions of the depressed patients. Furthermore, after antidepressant treatment, the elevated DMN connectivity in MDD patients could be normalized.^56^ In general, previous studies mostly agreed that depressed patients had hyper-connectivity in the DMN.^51, 53^

DMN hyper-connectivity has been ascribed to SN dysfunctions in several previous studies on MDD patients.^49, 51^ Increased communication between DMN and SN may have helped to form an aberrant circuit causing biased self-referential processes. To further test this theory, we extracted signals from the bilateral insula and mPFC, and calculated their correlations. The results showed that the coordination between the left insula and mPFC was significantly increased in the patient groups, despite that the overall connectivity strength was reduced. Therefore, our results support the theory that biased SN modulation on DMN contribute to depression.

There are several limitations in the present study. First, due to the heavy work load in our hospital, all of our patients were evaluated by only one psychiatrist, instead of two or more, through structured interviewing to diagnose MDD using the SCID (Structured Clinical Interview for DSM Disorders). Hence, some subjective bias might have been introduced during the diagnosis and scale evaluation procedure. Second, although our study had revealed brain network alterations in the patients group, the results were limited to the resting-state brain activities. How these brain areas activate and interact during emotional processing is important and need task-based fMRI investigations. The third limitation is the relatively small sample size. The findings of the present study still need further validation in a larger patient cohort.

In general, our study revealed reduced functional connectivity within several important nodes of emotional networks. These results are consistent with two main theories about brain network disruptions in MDD patients, and suggest that functional brain network had already been interrupted in the early stages of MDD. The ECM method has proven to be a powerful tool in detecting brain network changes, and is of great potential in studying the neuropathology underlying MDD, without the introduction of subjective ROI bias. Therefore, future studies using the ECM method to observe the brain network dynamics related to the development and treatment of MDD are warranted.

## Figures and Tables

**Table 1:** Demographic characteristics

	*Patients*	*Controls*	P
Age (years)	30.9±9.5	27.5±6.4	0.124
M/F	11/17	13/14	0.591
Duration (m)	5.5±3.5	—	—
HRSD	29.1±4.9	—	—
BDI	32.9±8.3	—	—

Abbreviations: BDI, Beck depression index; F, female; HRSD, Hamilton rating scale for depression; M, male.

**Table 2:** Areas showing significant difference of functional connectivity strength between the two groups

*Contrast*	*Voxels*	*Brain regions*	*MNI coordinate*	*Peak* t
NC>MDD	145	Left cerebellum	−21 −63 −30	7.18
	79	Right cerebellum	27 −63 −33	6.47
	35	Right hippocampus Right parahippocampal gyrus	21 −27 −9	5.60
	51	Left hippocampus Left parahippocampal gyrus Left amygdala	−30 −12 −15	5.23
	65	Right insula	30 18 −3	5.39
	22	Left insula	−33 3 −3	5.47
	12	Left middle frontal gyrus	−27 −6 42	5.21
	10	Right middle frontal gyrus	42 0 42	5.19
NC<MDD	241	Medial frontal gyrus	3 63 3	6.41

Abbreviations: NC, normal control; MDD, major depressive disorder.

**Table 3:** Frontal–subcortical connectivity and insula–mPFC connectivity in the two groups

	*NC*	*Depression*	P-*value*
*l_middle_frontal_gyrus*			
l_hippocampus_amygdala	0.69±0.29	0.34±0.22	0.000
r_hippocampus_amygdala	0.61±0.26	0.28±0.17	0.000
			
*r_middle_frontal_gyrus*			
l_hippocampus_amygdala	0.62±0.26	0.35±0.14	0.000
r_hippocampus_amygdala	0.57±0.23	0.25±0.18	0.000
			
*medial prefrontal cortex*			
l_insula	0.02±0.26	0.25±0.22	0.003
r_insula	0.05±0.28	0.14±0.22	0.15

Abbreviation: NC, normal control.

Ten comparisons had been made, and the threshold for significance is set at *P*=0.005.
